# Tuberculous Retroperitoneal Lymphadenitis After Kidney Transplantation That Was Difficult to Diagnose and Treat: A Case Report

**DOI:** 10.1002/iju5.70076

**Published:** 2025-09-01

**Authors:** Ryota Nakayasu, Yuki Kita, Yuki Teramoto, Kenji Nakamura, Toru Sakatani, Kimihiko Masui, Takayuki Goto, Ryoichi Saito, Takashi Kobayashi

**Affiliations:** ^1^ Department of Urology Kyoto University Graduate School of Medicine Kyoto Japan; ^2^ Department of Diagnostic Pathology Kyoto University Graduate School of Medicine Kyoto Japan

**Keywords:** abscess, extrapulmonary tuberculosis, immunosuppressive therapy, kidney transplantation, lymphadenitis

## Abstract

**Introduction:**

We present a rare case of tuberculous retroperitoneal lymphadenitis after kidney transplantation that was difficult to diagnose and treat.

**Case Presentation:**

A 52‐year‐old man who received a kidney transplantation from his wife presented with right lower quadrant pain. Computed tomography (CT) scan revealed multiple enlarged retroperitoneal lymph nodes, which later formed abscesses. From the positive *Mycobacterium tuberculosis* polymerase chain reaction test and smear in the drainage fluid, he was diagnosed with tuberculosis. Despite treatment with antituberculosis drugs, the abscesses increased, and he was treated by open drainage. He showed gradual clinical improvement and was discharged 9 weeks after hospitalization. While an abscess around the pancreatic tail remained, he was in remission, and antituberculosis drugs were terminated 404 days after initiation. He has gone 1 year without recurrence.

**Conclusion:**

Tuberculous lymphadenitis after kidney transplantation is a rare condition but should be kept in mind for accurate diagnosis.

**Summary**

- The incidence of tuberculosis infection after kidney transplantation is low, but it is important to diagnose it through a combination of appropriate tests and to carry out treatment patiently, with caution regarding side effects.

**Abbreviations**
CT: computed tomography
IL‐2R: interleukin‐2 receptor
MRI: magnetic resonance imaging
PCR: polymerase chain reaction
PTLD: post‐transplant lymphoproliferative disorder

## 1 Introduction

Tuberculosis occurs 20–50 times more frequently in kidney transplant recipients than in healthy individuals [1], with the majority of cases presenting as pulmonary disease. Tuberculous lymphadenitis following kidney transplantation is extremely rare. Here, we report a case of tuberculous retroperitoneal lymphadenitis after kidney transplantation, which posed difficulties in diagnosis and treatment.

## 2 Case Presentation

**FIGURE 1:**
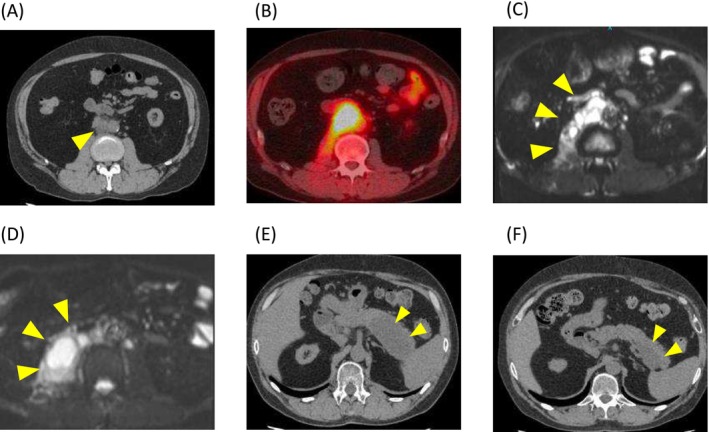
CT and MRI images. (A) At the time of initial presentation, enlarged retroperitoneal lymph nodes were observed. (B) 18F‐fluorodeoxyglucose positron emission tomography image showed increased fluorodeoxyglucose uptake in the retroperitoneal lymph nodes. (C) Post‐hospitalization MRI showed a multifocal abscess. (D) The abscess continued to increase after anti‐tuberculosis drugs were started. (E) Remaining abscess in the pancreatic tail was observed at the end of anti‐tuberculosis drugs. (F) The abscess continues to shrink even after the anti‐tuberculosis drugs have been terminated.

**FIGURE 2:**
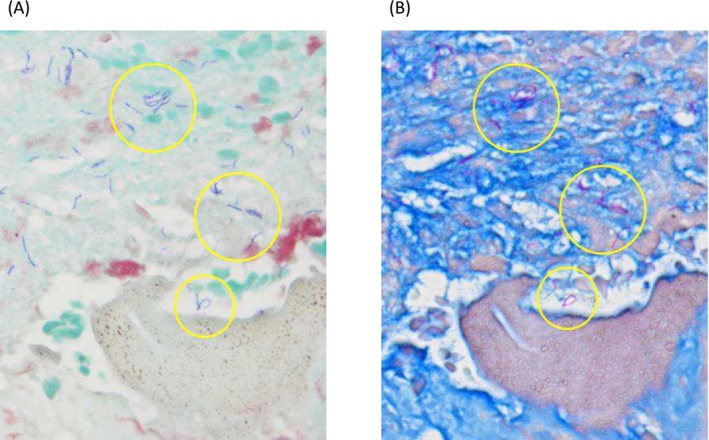
Gram staining and Ziehl‐Neelsen staining (A) Initial needle biopsy showed Gram‐positive rods. (B) The organisms diagnosed as Gram stain positive were positive for Ziehl‐Neelsen staining, which indicates that they are acid‐fast bacteria.

**FIGURE 3:**
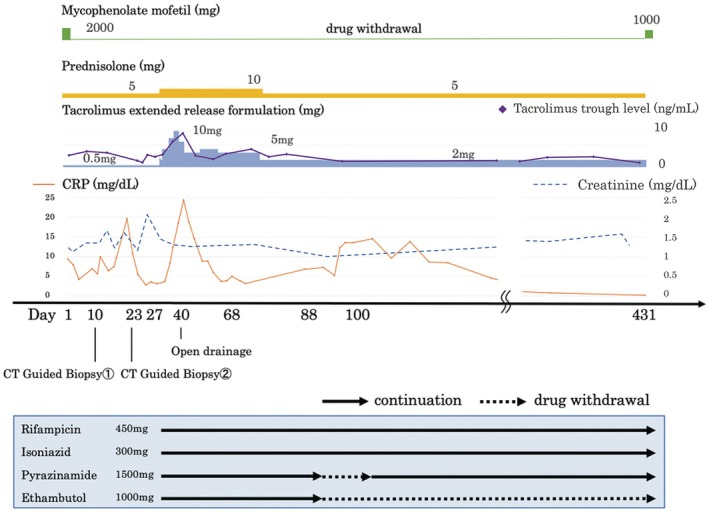
Dose adjustment of immunosuppressive drugs and anti‐tuberculosis drugs. Antituberculosis therapy with four drugs was initiated on Day 27 and reduced to two drugs on Day 88. Due to the presence of fever, the regimen was increased to three drugs on Day 100. Anti‐tuberculosis drug treatment was discontinued 404 days after it was started.

A 52‐year‐old man who had begun hemodialysis 8 years previously for chronic kidney failure due to diabetic nephropathy received a living‐related kidney transplantation from his wife. His preoperative tuberculin test was negative. He had been undergoing immunosuppressive management without rejection, with a combination of prednisolone (5 mg/day), tacrolimus (target trough level after 3 months posttransplantation: 4–6 ng/mL), and mycophenolate mofetil (2000 mg/day). Seven years later, he presented with right lower pain persisting for 1 month and underwent a CT scan, which revealed multiple enlarged retroperitoneal lymph nodes (Figure 1A). He was admitted for further examination and treatment. Soluble interleukin‐2 receptor (IL‐2R) was elevated at 1120 U/mL, and thus posttransplant lymphoproliferative disorder (PTLD) was suspected. F18‐fluorodeoxyglucose positron emission tomography CT scan showed accumulation in the lesion (SUV max = 9.0) (Figure 1B), consistent with PTLD, but CT guided biopsy showed Gram‐positive rods (Figure 2A). Given the patient's posttransplant immunosuppression and gram‐positive rods with beaded branching filaments, nocardiosis was suspected [2] and treated with teicoplanin, imipenem, and trimethoprim‐sulfamethoxazole. However, cultures were negative for Nocardia, and symptoms persisted despite antibiotics, suggesting alternative infections. MRI revealed multifocal abscesses suggestive of mycobacterial infection (Figure 1C). A repeat biopsy of the retroperitoneal lymph node confirmed tuberculous lymphadenitis, based on positive results from antimycobacterial smear and tuberculosis PCR. Positive Ziehl‐Neelsen staining on the initial biopsy specimen was also confirmed (Figure 2B). The right lower pain resolved after admission, and as there were no findings suggestive of PTLD despite the elevated IL‐2R levels, both were considered nonspecific reactions associated with tuberculosis. Four antituberculosis drugs were started but the patient's symptoms did not improve, and MRI showed an enlarged abscess (Figure 1D), so he was treated by open drainage. Under general anesthesia, the patient underwent retroperitoneal drainage of the abscess via a lumbar oblique incision. The procedure was completed by placing the tips of two drainage tubes within the abscess cavity. These drains were subsequently removed on postoperative Days 6 and 11, respectively. He gradually improved and was discharged on Day 68. Fever developed 1 week after reducing therapy to two antituberculosis drugs, but resolved following escalation to a three‐drug regimen. As the pancreatic tail cyst persisted without fever or CRP elevation, the infection was no longer active. Accordingly, antituberculosis therapy was discontinued on Day 404, and mycophenolate mofetil (MMF) was resumed. Notably, renal function remained stable throughout the period of MMF discontinuation (Figure 3). Subsequently, the cyst in the pancreatic tail gradually decreased in size (Figure 1F). The patient has been relapse‐free for 1 year since the treatment was terminated.

## 3 Discussion

The incidence of tuberculosis in kidney transplant recipients was reported as 0.42% and 0.45% in the USA and France, respectively, suggesting that tuberculosis after kidney transplantation is a rare disease [3, 4], while that is 20–50 times higher than in healthy individuals [1]. Extrapulmonary tuberculous lymphadenitis accounts for 5.5% of tuberculosis cases [5], but tuberculous retroperitoneal lymphadenitis after kidney transplantation has not been previously reported.

Mathieu et al. reported ring or multifocal enhancing effects in tuberculous lymph nodes in the abdomen [6], and the presence of multifocal abscess formation in this case could be used as a differential for tuberculosis. Tuberculosis is diagnosed based on characteristic histopathological findings or the detection of *Mycobacterium tuberculosis* by smear, culture, or PCR [7]. The diagnosis of tuberculosis can also be made with cytological specimens if all the following histopathological findings of tuberculosis are present: epithelioid cells, Langhans giant cells, and dry necrosis. However, these three findings appeared in the same specimen by puncture aspiration in only approximately 40% of cases [8]. The positivity rates of the antimicrobial smear test, antimicrobial culture test, and PCR test in the puncture aspiration method are 0%–62%, 19%–81%, and 47%–96%, respectively [8], so the positivity rate of these tests alone is not very high. In contrast, Beck et al. reported positivity rates of 52.9%, 76.4%, and 82.4% for histopathological findings, PCR tests, and their combination, respectively, suggesting that a combination of various tests is important [9]. In this case, absence of tuberculosis findings on initial biopsy and detection of Gram‐positive rods led to delayed diagnosis, initially suspected as nocardiosis. Mycobacteria are Gram‐indeterminate and can be Gram‐stain positive [10, 11]. Tuberculosis was considered as a differential diagnosis based on the patient's history and could only be diagnosed by adding specific stains. Tuberculosis sources include contact, donor‐derived transmission, or reactivation under immunosuppression [12]. In this case, there was no known contact history, and the preoperative tuberculin skin test was negative. Although the donor was not tested and false‐negative results are possible in renal impairment [13], both donor‐derived infection and reactivation were considered. Unrecognized exposure could not be completely ruled out.

Treatment of tuberculosis during immunosuppressive drug use consists of a four‐drug combination of rifampicin, isoniazid, pyrazinamide, and ethambutol for 2 months and rifampicin and isoniazid for 4 months [14]. Ogus et al. reported that antituberculosis drugs were required for 16 months for treatment of tuberculous lymphadenitis of the mediastinum and axilla after kidney transplantation [1]. In the present case, it took 13.5 months from the start to the end of antituberculosis drug treatment. Tuberculous psoas abscesses require both antituberculosis therapy and drainage. Although percutaneous drainage is standard, open surgery is appropriate for multiloculated abscesses or poor drug response [15]. In this case, open drainage led to clinical and inflammatory improvement and was considered an effective treatment strategy.

Adjustment of immunosuppressive therapy after the initiation of antituberculosis treatment is crucial. A caveat to long‐term continuation of antituberculosis drugs is that rifampicin induces the cytochrome P‐450 IIIA (CYP3A) enzyme. When combined with rifampicin, prednisolone should be doubled [16] and calcineurin inhibitors should be increased by checking blood levels, as their metabolism is accelerated and half‐life shortened with combination treatment. Inadequate blood levels of immunosuppressive drugs can lead to graft loss; Costa et al. reported graft loss because of tuberculosis after kidney transplantation in 14.7% of cases [16]. In the current patient, prednisolone was increased from 5 to 10 mg and the tacrolimus extended‐release formulation was increased from 0.5 to 5 mg (Figure 3), which was controlled within the intended blood levels in monitoring. Although the necessity of discontinuing MMF during tuberculosis treatment post‐transplant remains unclear, clinical improvement in post‐transplant testicular tuberculosis has been reported with mycophenolate mofetil discontinuation, antituberculosis therapy, and surgical drainage [17].

## 4 Conclusion

We report a case of tuberculous retroperitoneal lymphadenitis that developed 7 years after kidney transplantation, presenting diagnostic and therapeutic challenges. Tuberculous lymphadenitis after kidney transplantation is a rare condition, but it should be kept in mind as one of the differential conditions for enlarged lymph nodes.

## Ethics Statement

The authors have nothing to report.

## Consent

Informed consent was obtained from the patient, with guarantees of confidentiality.

## Conflicts of Interest

The authors declare no conflicts of interest.
